# A learning theory of meta learning

**DOI:** 10.1093/nsr/nwae133

**Published:** 2024-04-03

**Authors:** Fang Yao

**Affiliations:** School of Mathematical Sciences, Peking University, China

## Abstract

This paper gives a brief introduction to recent theoretical advance of meta learning.

A key feature of human intelligence is the ability to transfer knowledge learned from multiple learning tasks to other similar but different ones. Namely, humans attempt to learn how to generalize, and thus they can tackle a continuous stream of learning tasks. Correspondingly, in real-world applications, it is always crucial to improve learning in a new task by leveraging knowledge transferred from related tasks that have already been learned. Meta learning or learning to learn aims to tackle such a problem. As an exciting new research direction, there has been a significant interest in developing a theory to formulate meta learning.

The early works have mainly focused on meta learning via representation learning [1] or meta-representation learning [2]. Fixed representation or meta representation has insufficient flexibility that deals with changing task distributions. Recently, a simulating learning methodology (SLeM) approach [3] treats meta learning as learning an explicit hyperparameter prediction meta learner $h:\mathcal {T} \rightarrow \Psi$, mapping from the learning task space $\mathcal {T}$ to the hyperparameter space Ψ, which should potentially own better task generalizability than early focuses. Formally, one could use $\mathcal {LM}(D;\psi )$ to represent the extracted learner $f \in \mathcal {F}$ by implementing the corresponding machine learning process equipped with the hyperparameter configuration ψ ∈ Ψ by inputting the dataset *D* for the task at hand. This formulation allows one to apply standard statistical learning theory tools to investigate meta-learning theory. The following theorem presents the upper bound of the task transfer risk $\mathcal {E}(h) = R_{\eta }(\hat{h}) - R_{\eta }(h^{*})$ for SLeM meta learning, where $\hat{h}, h^{*}$ are the estimated and true meta learners, respectively.

**Theorem 1 [3].**

**Table 1.** Comparison of the main notations used in machine learning and SLeM meta learning [3].

Compared concepts	Machine learning	SLeM meta learning
Source distribution	Task distribution μ	Environment η
Training instance	Training dataset $D = \lbrace (x_i,y_i)\rbrace _{i=1}^n \sim \mu ^n$	Training task set $\boldsymbol {D} = \lbrace D_t = (D_t^{tr},D_t^{val})\rbrace _{t=1}^T$,
		training set $D_t^{tr}\sim (\mu _t^s)^{m_t}$, test set $D_t^{val}\sim (\mu _t^q)^{n_t}$,
		$\mu _t = (\mu _t^s,\mu _t^q) \sim \eta$
Test instance	(*x, y*) ∼ μ	$D_{\mu }^{tr}\sim (\mu ^s)^{m_{\mu }},\, (x,y) \in \mu ^q,\, \mu = (\mu ^s,\mu ^q) \sim \eta$
Learning objective	Learner $f:\mathcal {X}\rightarrow \mathcal {Y},\, f\in \mathcal {F}$	Meta learner $h:\mathcal {T} \rightarrow \Psi ,\, h \in \mathcal {H}$
Output of the (meta) learner	*y* = *f*(**x**; ω)	ψ = *h*(*T*; θ)
Performance measure	$R_{\mu }(f) = \mathbb {E}_{(x,y) \sim \mu } \ell (f(x),y)$	$R_{\eta }(h) = \mathbb {E}_{\mu \sim \eta }\mathbb {E}_{D^{val}\sim {(\mu ^q)}^n} \mathbb {E}_{D^{tr}\sim {(\mu ^s)}^m}$
		$\boldsymbol {L}(\mathcal {LM}(D^{tr}; h), D^{val})$
Goal of the generalization	Predicting label *y* for a query sample *x*	Predicting hyperparameter configurations ψ
		for a query task *T*

\begin{eqnarray*}
\mathcal {E}(h) &\lesssim & \mathcal {O}\bigg (\sqrt{\frac{C(\mathcal {F})}{m_{\mu }}}\bigg )+ \mathbb {E}_{\mu \sim \eta } d_{\mathcal {F}} (\mu ^s,\mu ^q) \\
&& +\, \alpha \tilde{\mathcal {O}} \bigg (\sqrt{ \frac{C(\mathcal {H})}{\sum _{t=1}^T n_t}}\\
&& +\, \frac{1}{T}\sum _{t=1}^T \bigg (\sqrt{ \frac{C(\mathcal {F})}{m_t}} + d_{\mathcal {F}} (\mu _t^{s},\mu _t^{q}) \bigg ) \bigg ).
\end{eqnarray*}

In Table 1 we list the main related notations of machine learning and SLeM meta learning, so that we can easily compare their main differences. As is well known, most procedures of conventional statistical machine learning are designed to learn a model from one single task/distribution. Differently, SLeM aims to construct a meta learner from multiple tasks (i.e. $\lbrace D_t = (D_t^{tr},D_t^{val})\rbrace _{t=1}^T$), and then transferably use it to help fulfill new test tasks μ ∼ η. In addition to the complexity of learning the task-specific learners $f \in \mathcal {F}$ for machine learning, SLeM contains a hyperparameter α that expresses the similarity between training tasks and test tasks, the complexity of learning the meta learner $h \in \mathcal {H}$ and the pre-defined divergence metric $d_{\mathcal {F}} (\mu _t^{s},\mu _t^{q})$ between support (training) and query (test) sets. The last metric especially tells us that the requirement of machine learning for the distribution of training and test sets to be consistent is not necessary for SLeM meta learning. All of these elements provide a fresh understanding and extension of conventional machine learning. Besides, similar to the structural risk minimization (SRM) principle in machine learning [4], it is hopeful that SLeM can develop proper meta-regularization techniques based on the proposed theory in Theorem 1 to enhance their generalization capability at the task level. This should be potentially beneficial to the meta-learning field, just like the significance of the SRM principle in the conventional machine learning field.

***Conflict of interest statement.*** None declared.
